# *Oligomeropathies*, inflammation and prion protein binding

**DOI:** 10.3389/fnins.2022.822420

**Published:** 2022-08-23

**Authors:** Gianluigi Forloni, Pietro La Vitola, Claudia Balducci

**Affiliations:** Department of Neuroscience, Istituto di Ricerche Farmacologiche Mario Negri IRCCS, Milan, Italy

**Keywords:** Alzheimer, Parkinson, amyloid, neurotoxicity, gliosis, oligomers

## Abstract

The central role of oligomers, small soluble aggregates of misfolded proteins, in the pathogenesis of neurodegenerative disorders is recognized in numerous experimental conditions and is compatible with clinical evidence. To underline this concept, some years ago we coined the term *oligomeropathies* to define the common mechanism of action of protein misfolding diseases like Alzheimer, Parkinson or prion diseases. Using simple experimental conditions, with direct application of synthetic β amyloid or α-synuclein oligomers intraventricularly at micromolar concentrations, we could detect differences and similarities in the biological consequences. The two oligomer species affected cognitive behavior, neuronal dysfunction and cerebral inflammatory reactions with distinct mechanisms. In these experimental conditions the proposed mediatory role of cellular prion protein in oligomer activities was not confirmed. Together with oligomers, inflammation at different levels can be important early in neurodegenerative disorders; both β amyloid and α-synuclein oligomers induce inflammation and its control strongly affects neuronal dysfunction. This review summarizes our studies with β-amyloid or α-synuclein oligomers, also considering the potential curative role of doxycycline, a well-known antibiotic with anti-amyloidogenic and anti-inflammatory activities. These actions are analyzed in terms of the therapeutic prospects.

## Introduction

The term “neurodegenerative disorders” covers pathologies with various epidemiological impacts and profound differences in their clinical manifestations variability and the selective vulnerability of neuronal systems might distinguish single diseases. In contrast with this, some pathological events driving the neuronal dysfunction and disease progression are common to virtually all neurodegenerative disorders. Protein misfolding and inflammation are a constant part of the neurodegenerative process although specific elements can characterize any single pathology. These common events generally precede the neuronal dysfunction and the involvement of other important elements responsible for the pathological processes. Protein aggregates are localized in the brain parenchyma, intra-neuronal or even inside the nuclei – as for huntingtin in Huntington’s disease (Sieradzan et al., 1999; Bates, 2003).

In Alzheimer’s disease (AD) the appearance of amyloid plaques is the first pathological event. However, the presence of β amyloid (Aβ) plaques does not correlate with the severity of the disease. Synaptic loss, the main feature associated with cognitive decline in AD, correlates better with tau neurofibrillary tangles, the other neuropathological hallmark of the disease (Savva et al., 2009; Seemiller et al., 2021; Stopschinski et al., 2021), and Aβ deposits are sometimes also seen in cognitively healthy subjects (Lue et al., 1999; Näslund et al., 2000). The presence of Aβ oligomers (AβOs), small soluble aggregates of misfolded protein (Kuo et al., 1996; Lue et al., 1999; McLean et al., 1999; Legleiter et al., 2010) might explain this as they are diffusible and exert potent neurotoxic activity (Haass and Selkoe, 2007; Niewiadomska et al., 2021). Oligomers contribute to plaque formation, while, the soluble aggregates are released from the deposited fibrils in a dynamic relationship (Gaspar et al., 2010; Shahnawaz and Soto, 2012; Katzmarski et al., 2020; Cascella et al., 2021; Ono and Watanabe-Nakayama, 2021).

A central pathological role of oligomeric conformers of misfolded protein has been proposed in other neurodegenerative disorders too, including, transmissible spongiform encephalopathies (TSE, Rezaei, 2008), Parkinson’s disease (PD) and Lewy bodies dementia (LDB) (Sokolowski et al., 2003; Gadad et al., 2011; Winner et al., 2011; Choi and Gandhi, 2018; Wells et al., 2021) and frontotemporal dementia (Makrides et al., 2003; Montalbano et al., 2020b).

Neuropathological observations have shown that the progression of the disease in AD may follow specific neuroanatomical pathways with prion-like propagation mechanisms. Similar progression has been observed in PD (Wakabayashi, 2020; Pyatigorskaya et al., 2021) and in taupathies (Mudher et al., 2017; Alyenbaawi et al., 2020). This propagation suggested that α-synuclein (α-Syn), tau but also huntingtin, superoxide dismutase 1, and several other proteins could move among cells, and once they reach a new cell could act as a seed by recruiting endogenous proteins, leading to the formation of larger aggregates (Goedert et al., 2010; Gerson and Kayed, 2013; Lee and Kim, 2015; Morozova et al., 2015; Forloni et al., 2016; Gerson et al., 2016; Marrero-Winkens et al., 2020). This evidence stems from clinical and experimental observations of a single cell, between nearby cells, and over longer distances throughout the brain (Klingelhoefer and Reichmann, 2015; Forloni et al., 2016; Brás and Outeiro, 2021; Guo et al., 2021) Thus PD/LBD and frontotemporal dementia can follow a propagation mechanism similar to AD or TSE although the seeding mechanism is intracellular (Danzer et al., 2009). Evidence from prion proteins suggests two dissociable mechanisms: intracellular toxicity and a non-toxic mechanism of propagation (Noble et al., 2015; Forloni et al., 2016). Both are exerted by oligomeric forms of misfolded proteins, and the term *oligomeropathies* correctly identifies protein misfolding-related diseases (Forloni et al., 2016).

## Oligomers and neurotoxicity

The amyloidogenic process described as protein polymerization into a beta sheet-rich conformer, insoluble, with a fibrillogenic ultrastructure, involves passages from oligomers to protofibrils and mature fibrils. Numerous factors including pH, ionic strength, temperature, mutations in amino acid sequence, metals, concentration, and incubation time, affect the amyloidogenesis. However, a common mechanism, regardless of the protein involved, seems to govern the formation of aggregates. Jarret and Lansbury (1993) proposed the seeding mechanism in the deposition of Aβ in AD and prion protein in TSE (Come et al., 1993), a nuclear-dependent polymerization in the formation of amyloid fibrils. *In vitro* amplification techniques for various amyloidogenic proteins have confirmed the central element of the seeded nucleation model.

Aβ plaques and oligomers in AD are generally formed by different Aβ peptides, the main components being the Aβ1–40 and Aβ1–42 sequences, and their self-aggregation capacity follows different kinetics: Aβ 1–42 spontaneously aggregates within minutes, while in similar conditions Aβ 1–40 takes hours or days to assemble in fibrils (Nirmalraj et al., 2020). Thus the seeding mechanism is activated by the highly fibrillogenic Aβ 1–42. Once the AβOs are formed, the biological effects are relatively independent from the initial sequences (Balducci et al., 2010; Forloni and Balducci, 2018). The N-terminally truncated pyroglutamylated form of Aβ (AβpE), also identified in AD brains with high self-aggregation capacity, has been indicated as the seed of nucleation of the Aβ 1–42 solution (Nussbaum et al., 2012; Pivtoraiko et al., 2015; Sofola-Adesakin et al., 2016).

The synergistic effect of Aβ/AβpE hetero-oligomers results in a species with a high level of toxicity, stabilizing the oligomeric structures and retarding fibril formation (Goldblatt et al., 2017). However, the deposition of the full-length or truncated forms of Aβ 1–42 shows a different distribution (Shinohara et al., 2017). Other Aβ peptide species in the brain are Aβ 1–43, and Aβ 1–37 or 1–38, and although experimental studies have used Aβ 1–42 solutions, the natural substrate of oligomers is a mixture of peptides. Amyloid deposition is considered an early pathological event in AD (Jack et al., 2013). As recently indicated by a meta-analysis, high levels of Aβ were associated with limited cognitive impairment and decline in cognitively normal older adults, suggesting a possible dementia prodromal condition (Baker et al., 2016; Forloni and Balducci, 2018; Mroczko et al., 2018).

Early studies analyzing the mechanism of polymerization focused on TSE, where the formation of small aggregates and the template role exerted by the pathological β sheet-rich form of prion protein (PrP*^sc^*) were associated with plaque deposition, with progressive distribution throughout the brain, but also with infectivity according to Prusiner’s hypothesis of DNA-free transmission (Prusiner et al., 1998).

The seeding mechanism has now been used for diagnostic purposes in TSE and other proteinopathies, to identify the tiny parts of pathological protein in biological fluids (Colby et al., 2007; Deleault et al., 2007; Caughey et al., 2017; Soto and Pritzkow, 2018; Iranzo et al., 2021) and nasal mucosa (Redaelli et al., 2017). Cyclic amplification of protein misfolding was initially proposed for the determination of PrP*^sc^* associated with TSE (Saborio et al., 2001). This was obtained by a series of incubations of the minimal parts of PrP*^sc^* with brain homogenate containing PrP*^c^* followed by sonication to generate multiple smaller units to favor the continued formation of new PrP*^sc^*, which became detectable with the classical methods.

Successively a new prion seeding assay was described called “real-time quaking-induced conversion assay” (RT-QuIC, Wilham et al., 2010) based on the possibility of amplifying the seed of PrP*^sc^*, with incubation in the presence of recombinant PrP and identifying the new aggregates with fluorescent thioflavine T. RT-QuIC has also been employed to identify α-Syn and tau signals in cerebrospinal fluid (CSF) and olfactory mucosa in various pathological conditions (Orrú et al., 2014; Kang et al., 2019; Saijo et al., 2019; Metrick et al., 2020; Perra et al., 2021; Stefani et al., 2021). The release of oligomers combined with the seeding mechanism might also be responsible for the wide spread of the pathology in neurodegenerative disorders. This has been well documented in synucleinopathies and taupathies (Goedert et al., 2014; Kovacs et al., 2014; Zhang et al., 2018; Hijaz and Volpicelli-Daley, 2020).

Independently of the amplification techniques described above, as soon as oligomers were proposed as key elements in the neurodegenerative disorders the search was on for oligomers in biological fluid and brain tissue (Roher et al., 1996; Gong et al., 2003; Dimant et al., 2013). Immunological and biochemical methods were developed although the multiplicity of the oligomeric species and their instability do not favor well-established, reliable methodology. In contrast with the monomeric form, a substantially higher AβO concentration in CSF was found in AD (Savage et al., 2014) and an increment of α-syn oligomers (α-synOs) was observed in PD (Tokuda et al., 2010; Parnetti et al., 2019). The detection of oligomers in CSF was proposed as diagnostic tool (Wang-Dietrich et al., 2013; Majbour et al., 2020). The presence of specific oligomer species (Aβ*56, dimers and trimers) was investigated, seeking the correlations with pathological or pre-pathological conditions (Lesné et al., 2013) and with neuropathological events (Klyubin et al., 2008; Handoko et al., 2013) with interesting but not always replicable.

Recently, using surface-based fluorescence intensity distribution analysis (sFIDA) α-SynO and tau oligomers (TauO, Blömeke et al., 2022) and AβO (Kass et al., 2022) have been accurately determined. The concentration and size distribution of AβOs were determined in AD brain samples using sFIDA in conjunction with density gradient centrifugation. The advantage of this approach lies in its ability to keep all the aggregates in a native state (Bhatt and Kayed, 2022). This technique substantially confirms the possibility of discriminating the different pathologies by determining the various oligomeric species.

In blood the levels of AβO (Zhou et al., 2012) and α-synO (Wang et al., 2015a) were altered in AD and PD, respectively, and were proposed as diagnostic tools. However, although the measurement of markers in blood samples is an interesting approach for the sampling accessibility, we are far enough from a well-established methodology and reliable results to consider these determinations as diagnostic tool.

As illustrated by Cascella et al. (2022) the detection of oligomers *in situ* is complicated by the lack of methods to detect these species specifically *in vivo* and in patient tissues, although interesting results have been obtained with α-syn proximity ligation assay (Roberts et al., 2015). Using mouse anti-α-syn antibody appropriately conjugated with activated oligonucleotides by Duolink^®^ kits, in medulla, midbrain and cingulate cortex from PD subjects, abundant amounts of oligomers were demonstrated (Roberts et al., 2015). Different α-synO species were found by proximity ligation assay in multiple system atrophy (MSA) brain (Sekiya et al., 2019). Heterogeneous distribution of α-synO species in MSA has been recently confirmed by Martinez-Valbuena et al. (2022) with the seeding technique. A few other investigations have been made on AβO and tau aggregates in brain tissue (Lasagna-Reeves et al., 2014; Savioz et al., 2016) but we are still waiting for systematic investigations on the oligomeric localization in human brain tissue associated with different pathologies. The conditions to identify oligomeric species in experimental models are more favorable. For instance in transgenic mice the detection of human oligomeric species is not influenced by the artifacts deriving from the endogenous monomeric forms during purification. Numerous treatments reduce the presence of cerebral oligomers and this could form part of future therapeutic strategies in neurodegenerative disorders (Santamaria et al., 2021; Kass et al., 2022).

Although it was initially postulated that Aβ the peptides’ neurotoxicity was closely related to their fibrillogenic capacity (Yankner et al., 1990; Lorenzo and Yankner, 1994) we demonstrated that the amidated form of Aβ peptide lost its fibrillogenic capacity but maintained the neurotoxic effect in primary hippocampal cultures (Forloni et al., 1997). This was part of the earliest evidence that the neurotoxicity of Aβ peptides was induced by a soluble form of aggregates rather than structured fibrils. This concept was further elaborated by Lambert et al. (1998) and the term oligomers associated with neurotoxicity emerged a couple of years later (El-Agnaf et al., 2000).

In studies *in vivo* described by Walsh et al. (2002), oligomers purified from human cell lines directly injected into the rat brain strongly inhibited long-term potentiation (LTP); these findings originally proved that oligomers could induce neuronal dysfunction. In pyramidal neurons in rat organotypic slices exposure to a picomolar concentration of oligomers induced progressive loss of hippocampal synapses and reduced dendritic spine density. Spine loss was reversible and was prevented by Aβ-specific antibodies or a small-molecule modulator of Aβ aggregation. The study further showed that Aβ-mediated spine loss required the activity of NMDA-type glutamate receptors (NMDARs) and occurred through a pathway involving cofilin and calcineurin (Shankar et al., 2007). These and many other experimental results confirmed the specific neurotoxic activity of AβOs through a reversible mechanism. The neuronal cell death caused by AβOs was reconsidered as a reversible neuronal dysfunction (Holscher et al., 2007) which, applied continually, might eventually induce irremediable neurodegeneration.

*In vivo* studies have identified specific oligomer species (Aβ*56) that can trigger the neuropathological sequences in experimental models when injected intracerebrally (Lesné et al., 2006). In humans cerebral Aβ*56 has been detected in relation with aging, tau phosphorylation and changes of some post-synaptic proteins before the appearance of cognitive decline (Lesné et al., 2013). This information was given little weight outside of the scientists involved in the original research (Amar et al., 2017) and was discussed in terms of methodological difficulties to get a reproducible protocol to determine Aβ*56 (Benilova et al., 2012).

The origin and size of the oligomer species, ranging from 8 to 200 KDa, and the cellular pathways proposed to mediate their effects vary widely (Benilova et al., 2012; Bobo et al., 2017; Ashe, 2020; Niewiadomska et al., 2021). The size of the oligomers can influence their biological activities although AβO preparation species can be found that are neither toxic nor recognized by oligomer-specific antibodies, indicating that AβOs may also present size-independent differences in toxicity, and differences may be seen between two AβOs of similar size and dissimilar toxicity (Ladiwala et al., 2012). The recent distinction between the toxicity of oligomers according to their aggregation size and the two way connections between the large and small oligomers confirmed the complexity and the non-linear trend of AβO formation (Pani et al., 2020). Studies suggest that the toxicity of small AβOs is governed primarily by the degree of solvent exposure of hydrophobic residues and is weakly influenced by their secondary structures (Bemporad and Chiti, 2012). Chaperones play an important part not only in the oligomer conformation and assembly but also in their biological interaction. Taking the size of oligomers and their hydrophobic exposure as the main determinants of their neurotoxicity (Mannini et al., 2014; Arosio et al., 2016; Lee et al., 2018) molecular chaperons increase in size and mask hydrophobic patches exposed on their surface (Mannini and Chiti, 2017). In humans, the diffuse presence of AβOs may possibly either precede or follow plaque formation; the variable production of toxic species and the neuronal vulnerability can differentiate the dynamics of the pathological process and the functional consequences (Forloni and Balducci, 2018; Madhu and Mukhopadhyay, 2021).

We developed a simple method to assess the effects of AβOs *in vivo* by direct intracerebroventricular (ICV) injection of a 1 μM solution followed by behavioral tests, histological and biochemical examinations. We realize that this approach has limitations considering the complexity of AD, but in this condition the biological effects of AβOs can be analyzed with no other confounding factors (Balducci and Forloni, 2014; Forloni and Balducci, 2018). Constant analysis of the injected product by atomic force microscopy (AFM) and the use of Aβ-42 depsipeptide minimize the variability in the structural conformation of AβOs and the initial state of the solution. Synthesis of the Aβ-42 depsipeptide includes the formation of an ester bond between the side chain hydroxyl group of serine-26 and the next amino acid glycine, using depsipeptide building blocks (Beeg et al., 2013). The resulting depsi-Aβ1–42 is highly water-soluble and adopts and retains a monomeric unordered state under acidic conditions and when the solution is stored at 80°C for several days (Taniguchi et al., 2009). The native Aβ1–42 sequence is then obtained easily and irreversibly from the depsipeptide by an O–acyl shift (or migration) under neutral conditions (Taniguchi et al., 2009). This conversion is very rapid and quantitative, and produces Aβ1–42 with an unordered structure that undergoes the usual oligomerization and amyloid fibril formation. Switching the depsipeptide means its aggregation can be triggered only when required (Beeg et al., 2013).

As our own experience and numerous studies indicate, the preparation of oligomers is a tricky step that needs to be closely controlled to guarantee reproducible results (Lasagna-Reeves et al., 2010; Forloni and Balducci, 2018). AβO 1–42 injections induced a memory impairment in the novel object recognition test (NORT). This cognitive test is based on the spontaneous behavior of the animals without specific training, and to minimize interference due to anxiety we tested the mice in their own cages. The toxic effect of AβOs was specific: Aβ 1–42 in monomeric or fibrillar form did not influence cognitive behavior; Aβ-antibody completely antagonized the AβO activity (Balducci et al., 2010).

Together with Aβ plaques, AD is neuropathologically characterized by accumulation of insoluble tau aggregates, defined tauopathy. In AD, this is considered secondary, while in primary tauopathies the neurodegenerative pathology is driven by tau deposition (Chung et al., 2021). In human brain, tau exists in six isoforms, which are mainly distinguished by the presence or absence of the second microtubule-binding repeat, R2, combined with other microtubule-binding repeats (R1, R3, R4). Tau hyperphosphorylation formed intracellular aggregates in various pathological conditions, the combination of different isoforms characterized the aggregation in the primary or secondary tauopathies. A recent study using solid-state nuclear magnetic resonance (NMR) spectroscopy established that in AD tangles R3 and R4 isoforms are combined with no structural differences between filaments with a single isoform or the combination of both (Dregni et al., 2022).

Growing evidence indicates that TauOs play a role in the pathogenesis of neurological diseases (Spires-Jones et al., 2009; Guerrero-Muñoz et al., 2015). Although the molecular size of tau protein species in monomeric form (about 60 kDa) is several times bigger than Aβ or α-Syn (4 and 14 kDa, respectively), the oligomerization mechanism, the heterogeneity of the species assembled (Kjaergaard et al., 2018) and the cerebral propagation of the soluble TauOs are similar to the aggregates deriving from other proteins. Relatively small TauOs have toxic activity in various conditions (Lasagna-Reeves et al., 2010; Fox et al., 2011; Flach et al., 2012; Niewiadomska et al., 2021) and intracellular and extracellular detrimental activities have been shown in experimental models (Pampuscenko et al., 2021). Exposure to TauOs affects memory and its cellular correlate long-term potentiation (LTP) (Lasagna-Reeves et al., 2011; Fá et al., 2016). Large amounts of TauOs are found in AD brains (Maeda et al., 2006) and tau pathology correlates well with disease progression and cognitive deficits. TauOs act on various levels: (i) in the nucleus, affecting the expression of genes related to synaptic damage (Violet et al., 2015); (ii) at the synapses, altering the expression of synaptic proteins, impairing synaptic plasticity and memory (Bolós et al., 2017).

In the cell nuclei tau exerts neuroprotective activity, interacting with DNA regions of the chromosome (Sjöberg et al., 2006; Sultan et al., 2011), so tau oligomerization may cause a loss of physiological function (Benhelli-Mokrani et al., 2018). The progressive increase of TauOs in the nucleus compartment is associated with a reduction in the ability to bind DNA (Benhelli-Mokrani et al., 2018; Niewiadomska et al., 2021), while on the other hand the TauOs may actively destabilize the DNA structure (Montalbano et al., 2020a). Furthermore, in AD, TauO assembles with RNA-binding protein Musashi (MSI) (Montalbano et al., 2019) and this association has been observed in brain tissue in different pathological conditions, in tau knock-out and P301L mouse animal models. The tau and MSI interaction alter the nuclear cytoplasm transport chromatin remodeling and the formation of lamina (Montalbano et al., 2019). The complex pathological activities associated with TauO formation in nucleic compartment have consequences at different levels and might be considered a therapeutic target in neurodegenerative disorders.

Alpha-synuclein, a 140 residue protein expressed abundantly in the brain, is the main component of intracellular inclusions, Lewy bodies and Lewy neurites, which neuropathologically characterize PD and related disorders (Spillantini et al., 1998). In Lewy bodies α-Syn is structured in fibrillar form with a high β-sheet component similar to Aβ. Alpha-syn, initially identified as the non-amyloid component of senile plaques (NACP, Weinreb et al., 1996) has a self-aggregation capacity similar to Aβ, with a slow kinetic influenced by mutations associated with PD (Conway et al., 1998). The nucleation polymerization mechanism is compatible with the formation of α-syn oligomers and fibrils (Wood et al., 1999).

Alpha-syn defined naturally unfolded protein can bind to lipid membrane, and has been described physiologically in alpha helical tetramer structures resistant to aggregation (Bartels et al., 2011; Wang et al., 2011; Xu et al., 2019). It has been suggested that tetramers undergo destabilization of their helically folded conformation prior to α-syn aggregation into abnormal oligomeric and fibrillar assemblies. The process of α-syn aggregation is complex but largely explained by the nucleation-polymerization model or nucleation-conversion-polymerization where the β-sheet structure is progressively incremented (Cremades et al., 2012, 2017; de Oliveira and Silva, 2019; Giampà et al., 2021). As in AβO formation, α-syn oligomerization is a heterogenous process in terms of size, from dimers to higher order multimers, structure, different β-sheet contents and ultrastructural differences, and transitory nature (Cremades et al., 2017; Guerrero-Ferreira et al., 2020). Alpha-syn fibril polymorphism has been ascertained by cryo-electron microscopy but a clear understanding of the structure of α-synOs is still lacking (Li et al., 2018; Du et al., 2022). The antiparallel intermolecular β-sheet structure has been observed in a stable, particularly toxic oligomeric form of α-syn and has been proposed as distinctive of α-synOs toxic species (Celej et al., 2012; Cascella et al., 2022).

Structural analysis of the interaction of α-synOs with lipid bilayers leading to cytotoxicity indicates that amphipathic alpha helics in the N-terminal region are important to exert membrane disruption (Fusco et al., 2017), and in agreement with this the antibody targeting N-terminal of α-syn greatly reduces the toxicity of oligomers (Lorenzen et al., 2014). Similarly to AβOs and other oligomeric species the core of α-synOs rich in β-sheet structure can insert lipid bilayers and destroy membrane integrity and can form pores in lipid bilayers and their β-sheet structure (Kim et al., 2009). However, this mechanism only partially accounts for the α-synOs neurotoxic effects, Cascella et al. (2021) showed that fibrils bound to the cell membrane did not correlate with the degree of cell dysfunction or α-syn penetration. The mitochondrial model membranes exposed to α-synOs are more vulnerable to permeabilization than these reconstituted from brain-derived lipids (Stefanovic et al., 2014). This interesting observation is important in the context of PD pathogenesis where mitochondrial damage is considered a prominent feature of the disease and the protection from mitochondrial alterations induced by α-synOs is a potential therapeutic target (Caruana et al., 2021).

In terms of pathogenic profile, the main difference between Aβ and α-syn aggregation is their compartmentalization: Aβ plaques in brain parenchyma, Lewy bodies intracellular, although, as noted before, α-syn fragments were found in Aβ plaques. Thus the pathogenic mechanisms in synucleinopathies imply the intracellular accumulation of α-syn, the passage from cell to cell (Desplats et al., 2009) and the neuronal dysfunction resulting from exposure to neurotoxic oligomeric species though in some conditions, α-syn fibrils too can induce neurotoxicity (Peelaerts et al., 2015; Tapias et al., 2017; Guiney et al., 2020; Zadali et al., 2021).

In the past we showed selective dopaminergic neurotoxicity with α-syn peptide (NAC) associated with TAT sequence to vehicle the peptide inside the cells (Forloni et al., 2000). The evidence from the determination of α-syn in CSF and other biological fluids (Borghi et al., 2000; El-Agnaf et al., 2003) and the possibility that Lewy bodies pathology might pass from host to grafted tissue (Kordower et al., 2008; Li et al., 2008) then completely changed the pathological hypothesis related to synucleinopathies that became compatible with a fundamental role of diffusible aggregates. Intracellular α-syn aggregation can be triggered by the introduction of recombinant α-syn fibrils conveyed by liposome into cultured cells overexpressing α-syn (Luk et al., 2009). Alpha-syn fibrils “seeded” recruitment of endogenous soluble α-syn protein and their conversion to insoluble, hyperphosphorylated, and ubiquitinated pathological species (Luk et al., 2009). Similar results have been obtained in SHSY-5Y cell lines exposed to simple α-syn short amyloid fibrils inducing intracellular aggregation (Aulić et al., 2014) and α-synOs internalized trough an active process like endocytosis (Oh et al., 2016; Shearer et al., 2021). Alpha-synOs is released from neuronal cells by non-conventional exocytosis involving extracellular exosomes, α-synOs associated to exosome can be also internalized via an endocytic pathway (Emmanouilidou et al., 2010; Delenclos et al., 2017).

After the initial studies on α-syn neurotoxicity (El-Agnaf et al., 1998; Lücking and Brice, 2000; El-Agnaf and Irvine, 2002) the toxicity of multiple α-synOs conformers was investigated in various conditions (Outeiro et al., 2008; Brown, 2010; Jiang et al., 2010; Celej et al., 2012) and the different species distinguished biological activities (Danzer et al., 2007). In this study, following three different protocols various species of α-synOs were formed, and heterogeneity was evident in all preparations. The biological activities of these aggregates were compared in in SHSY-5Y cells and primary culture in terms of effects on cell death, cytosolic calcium levels and intracellular α-syn aggregation. Smaller and annular structures affected calcium influx and cell death while larger structures exerted no neurotoxic activity but these oligomers could enter the cell directly and promote α-syn aggregate formation, as described by Lee et al. (2002). More recently Cascella et al. (2021) showed that in addition to the well-established contribution of α-syn fibrils to the diffusion of the pathology by a spreading mechanism (Danzer et al., 2012), the fibrillar species can have an immediate toxic effect due to the release of toxic oligomeric species in different cellular models, including human iPSC-derived dopaminergic neurons, rat primary cortical neurons, and human SH-SY5Y neuroblastoma cells (Bigi et al., 2021; Cascella et al., 2021). In iPSC model of synucleinopathies Prots et al. (2018) showed that according to the pathological progression of events in neurodegenerative disorders α-synOs caused axonal dysfunction. The application of the α-syn oligomerization model by expressing α-syn oligomer-forming mutants (E46K and E57K) and wild-type α-syn in human iPSC-derived neurons led to impaired anterograde axonal transport of mitochondria, which was restored by inhibiting α-synO formation (Prots et al., 2018). The exogenous application *in vivo* of α-synOs by ICV injection induced LTP inhibition and memory deficits through mechanisms that involve calcineurin activation (Martin et al., 2012). In a similar model α-synOs caused olfactory dysfunction and dopaminergic neurotoxicity in olfactory bulb, late motor deficits and striatal dopamine loss in mice (Fortuna et al., 2017). More recently, intranigral injections of α-synOs induced dopaminergic cell loss, an inflammatory reaction, motor and cognitive impairment – the basic pathological PD features (Boi et al., 2020).

**FIGURE 1:**
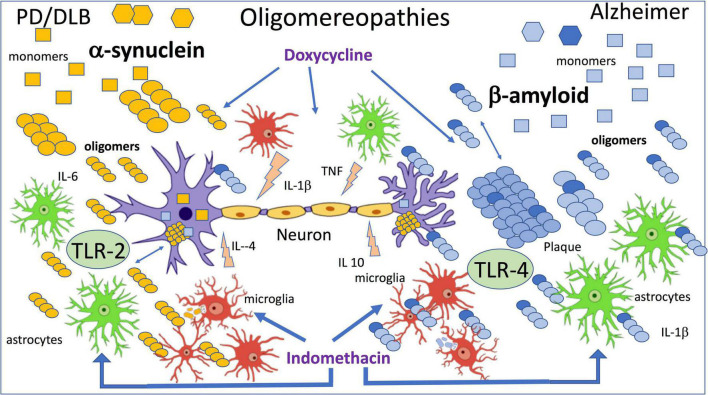
The figure summarizes the effects of β amyloid and α-synuclein oligomers directly applied by intracerebral injection. Wild-type mice were injected ICV with 7.5 μl of 1 μM oligomers, and indomethacin and doxycycline were injected peripherally 2 h before oligomers injection (Forloni et al., 2016; Balducci et al., 2018; La Vitola et al., 2018, 2019).

Using the method described above, we injected naïve mice with α-synOs and similarly to AβOs, these significantly impaired performance in the NORT, while the monomeric form and α-syn fibrils were not active. The effect was antagonized by an anti-α-syn antibody. These results were associated with inhibition of LTP in brain slices by application of α-synOs (La Vitola et al., 2018). As already proposed by Forloni et al. (2016) the differences and similarities characterizing the exposure to AβOs or α-synOs (Figure 1) were later confirmed in transgenic mice and other experimental models (La Vitola et al., 2021).

In line with previous findings (Rosenberger et al., 2014) and in association with activation of the inflammatory cascade, we investigated the role of toll-like receptors (TLR) in AβOs and then in α-synOs toxicity, focusing on TLR-2 and 4. The TLR-4 antagonist abolished the behavioral effect of AβOs injected in the brain and in agreement with this AβOs were no longer toxic in TLR-4-knockout mice (Balducci et al., 2017; Balducci and Forloni, 2020). Neither the pharmacological interference with TLR-4 nor their ablation affected the cognition impairment induced by α-synOs, whereas this activity was sensitive to the TLR-2 antagonist (La Vitola et al., 2018). There are numerous reports of a direct or indirect association of α-synOs with TLR-2 in different cell types responsible for the glial activation, although TLR-2 has also been expressed by neurons (Kwon et al., 2019; Dutta et al., 2021).

## Oligomers and inflammation

In the neurodegenerative disorders, together with the cerebral accumulation of misfolded proteins neuroinflammation has emerged as a common driving force of neuronal dysfunction in the early phase (McManus and Heneka, 2017; Forloni and Balducci, 2018; Meraz-Ríos et al., 2019). The positive activity of surveillance to eliminate the debris and the pathogenic elements resulting from phagocytosis of activated microglia and astrocytes can in certain circumstances be associated with the production of factors that harm the neuronal system (De Simoni et al., 1995; Schwartz and Deczkowska, 2016; Ceyzériat et al., 2018). Persistent activation of glial cells that compromises neuronal functionality can result from continuous exposure to an inflammatory stimulus or impairment of resolution (Chamani et al., 2020). Both mechanisms are present in AD and lead to persistent chronic inflammation. The immune response resolved after removal of the stimulating pathogen by the passive dissipation of the inflammatory mediator, but resolution is also an active process regulated by specialized pro-resolving mediators (SPMs) and resolvins, that promote the return to homeostasis by limiting inflammatory signals and suppressing the natural response during inflammation (Serhan et al., 2007; Serhan, 2014; Chamani et al., 2020; Miyazawa et al., 2020). This mechanism seems to be altered in AD, since recent studies identified receptors for SPMs in the human brain, low levels of SPMs in the brains of AD patients, and a correlation between lower SPM levels and cognitive impairment (Wang et al., 2015b). Furthermore, SPMs promote neuronal survival and increase the microglial phagocytosis of Aβ (Zhu et al., 2015, 2016). The neuroinflammation in AD persists, with no resolution, as a sort of permanent activation of the innate immune system that causes neuronal alterations (Zhu et al., 2018). This chronic inflammation can be associated with the continued presence of Aβ aggregates (AβOs). The altered equilibrium between inflammation and resolution has also been proposed in the pathogenesis of PD (Tian et al., 2015; Krashia et al., 2019). In our model AβOs or α-synOs injected ICV induced microglial and astroglial activation determined biochemically and histologically in the hippocampus and cortex with increases in the expression of IBA-1 and GFAP, respectively, within 24 h of the injection (Balducci et al., 2017; La Vitola et al., 2018, 2019).

Toll-like receptors, particularly TLR-2 and TLR-4 are the main mediators of inflammation (Kollmann et al., 2012; Trotta et al., 2014; Saleh et al., 2021) and in neurodegenerative disorders their activation has been associated with either harmful or beneficial effects, although recent data mainly support the negative action (Lehnardt et al., 2006; Leitner et al., 2019; Balducci and Forloni, 2020; Yang et al., 2020). As mentioned above, in our model measuring the cognitive decline we distinguished a role of TLR-4 in the AβOs and TLR-2 in the α-synOs toxic effect. Similarly, glial activation induced by AβOs was abolished by the TLR-4 antagonist or nullified in TLR-4-KO mice, while the effect of α-synOs was not altered by TLR-4 interference but was antagonized by the TLR-2 antagonist (La Vitola et al., 2018).

It is interesting that a relatively simple method such as direct intracerebral application of different oligomers can give information on the distinct mechanisms of action of common biological effects. There is growing evidence of TLR-4 as the mediator of the inflammatory mechanism in AD experimental models associated with Aβ (Liu et al., 2020). Aggregated Aβ binds to TLR-4 and activates microglia, resulting in increased phagocytosis and cytokine production. Inflammatory factors including TNF-α, interleukin 1β and interleukin 6 are under the nuclear factor kappa B (NFκB) transcription factor sensitive to TLR4-activation through a MyD88 dependent and independent pathway (Rahimifard et al., 2017; Cui et al., 2020; Hughes et al., 2020). TLR-4 was upregulated in the brains of AD patients and an AD mouse model (Walter et al., 2007; Figure 1). It was recently shown that trilobatin a flavonoid isolated from *Lithocarpus polystachyus* Rehd, trilobatin, had a neuroprotective effect In a murine model of AD (3×FAD) through TLR-4 inhibition (Ding et al., 2021). In that study the interference with TLR-4 attenuated all the negative events associated with the AD model phenotype: Aβ burden, neuroinflammation, tau hyperphosphorylation, synaptic degeneration, hippocampal neuronal loss, and memory impairment (Ding et al., 2021). This and several other similar findings indicate that targeting TLR-4 might be an interesting therapeutic approach to AD (Rahimifard et al., 2017; Paudel et al., 2020; Connolly et al., 2021). With polyphenols, several other agents have been identified as active against TLR-4, with neuroprotective activities in various experimental models (De Paola et al., 2012; Feng et al., 2017; Wang et al., 2019; Zhou et al., 2020; Connolly et al., 2021) but no data are yet available at the clinical stage, where novel tools modulating inflammation are still needed (Forloni, 2020).

We have seen that doxycycline, a tetracycline with optimal blood-brain barrier passage and a good safety profile, can be repurposed in neurodegenerative conditions. The anti-amyloidogenic activity of doxycycline demonstrated in the past (Forloni et al., 2001, 2002; Stoilova et al., 2013; Medina et al., 2021) has been associated with anti-inflammatory and anti-oxidant activity (Balducci and Forloni, 2019). We showed that systemic treatment with doxycycline antagonized the deleterious effects of intracerebral application of AβOs in terms of memory impairment and glial reactivity (Balducci et al., 2018). In APP/PS1 transgenic mice acute and chronic treatment with doxycycline had beneficial effects on short-term memory in the NORT, although only after a lengthy exposure this effect was associated with a reduction in Aβ plaques (Balducci et al., 2018). These results were consistent with direct interference with AβOs by doxycycline, that finally lead to reduced plaque formation. However, the drug antagonized the LPS-inducing cognitive decline in the absence of AβOs, confirming the anti-inflammatory activity described in numerous other experimental models (Zhang et al., 2015; Wiggins-Dohlvik et al., 2016; Mello et al., 2021).

As mentioned above, parallel studies with α-synOs injected ICV have similarities with AβOs but also some clear differences, (Figure 1) as reported by La Vitola et al. (2018). TLR-2, involved in the reversible neuronal dysfunction and glial activation induced by α-synOs is consistent with findings suggesting a key role for TLR-2 in synucleinopathies. The biological activity of α-syn in various forms was associated with TLR-2 in the CNS and periphery (Dutta et al., 2021; Scheiblich et al., 2021; Sun et al., 2021a; Xia et al., 2021). It has also been shown that α-synOs released by neurons can be considered endogenous agonists of TLR-2 on microglial cells (Kim et al., 2013) and later the same group showed that the inactivation of TLR-2 induced autophagy and increased the clearance of α-synOs in transgenic mice and *in vitro* models. These results indicate the importance of TLR-2 in regulating neuronal autophagy (Kim et al., 2015). They are summarized in a review by Kwon et al. (2019) where the interaction between α-synOs and TLR-2 is developed on three levels: microglia and astrocytic activation; the regulation of neuronal autophagy and the neuron to neuron and neuron to glial α-syn transmission. The authors proposed targeting TLR-2 as a promising immunotherapeutic approach to synucleinopathies (Kim et al., 2018; Kwon et al., 2019; Sun et al., 2021b). Similar conclusions were reported by Kouli et al. (2019) where the effect of TLR-2 restricted to the CNS was associated with an important role of TLR-4 played peripherally in synucleinopathies. A strong affinity of α-synOs for TLR-2 in beta-rich species was observed by Kumari et al. (2020).

We found interesting evidence when ICV α-synOs were given one month after a single peripheral injection of lipopolysaccharides (LPS), to establish lasting level of general inflammation. A sub-active dose of α-synOs synergized with the LPS to induce cognitive decline and microglial activation, while the combination of central application of α-synOs at low dosage and LPS did not enhance astroglial reactivity and in fact resulted in astrocyte dysfunction (La Vitola et al., 2021). Similarly, when α-syn A53T transgenic mice were given LPS, memory impairment and microglial activation were exacerbated, while the astrocyte activation was turned off. These data illustrate the influence of general inflammation on central α-synOs activity and the different reactivity of astrocytes and microglia to double-hit stimulation (La Vitola et al., 2021). Both findings are important to orient the therapeutic strategy in PD and associated synucleinopathies; the control of general inflammation might influence progression in the early phase of the disease and the astrocyte dysfunction is part of the pathological events associated with the activity of α-synOs (Chen et al., 2021; Wang et al., 2021).

Sensitivity to doxycycline is another common element shared by α-synOs and AβOs biological activities (Figure 1). In the last few years the interaction between α-synOs and doxycycline has been studied at different levels (González-Lizárraga et al., 2017; Bortolanza et al., 2018), and the drug’s anti-amyloidogenic and the anti-inflammatory activity both turned out to be essential to contrast the pathological spread in PD and related diseases (Dominguez-Meijide et al., 2021; Forloni et al., 2021). It is interesting to note that doxycycline can change the structure of α-synOs into off-pathway, high-molecular-weight species, that do not evolve into fibrils (González-Lizárraga et al., 2017). The incubation of α-syn in the presence of doxycycline did not interfere with the initial α-synOs formation analyzed by electron microscopy (EM), but when the incubation was continued, the oligomers did not evolve in fibers as in the control conditions. Furthermore, the α-synOs from incubation with doxycycline in the seeding experiment did not induce fibril formation.

The structural diversity in α-synOs exposed to doxycycline has been proved by small-angle X-ray scattering (SAXS), bis-ANS fluorescence, and Fourier Transformed Infrared spectroscopy (FT-IR). SAXS reported changes in the protein aggregates’ morphology.

Finally, the α-synOs deriving from incubation with doxycycline (off pathway) were minimally toxic compared to high toxicity of α-synOs (on-pathway) shown in SH-SY5Y cells, and the addiction of doxycycline in cell incubation medium strongly antagonized the toxicity (González-Lizárraga et al., 2017). Thus, as proved in several circumstances, doxycycline can prevent fibrils formation and disrupt fibrils already formed (Tagliavini et al., 2000).

The multi-target activities of doxycycline in different experimental conditions are shared by several other compounds, mainly of natural origin (Wu et al., 2019; Ahmad et al., 2021; Maccioni et al., 2022). Rifampicin, another well-known antibiotic that independently of its anti-infectivity action has an important neuroprotective function: anti-AβOs, anti-TauOs and anti-inflammatory activities have been demonstrated by rifampicin in various experimental models (Umeda et al., 2016) with a mechanism similar to that described for doxycycline (Yulug et al., 2018). A direct effect on α-synOs has been also shown in PD and LBD models (Acuña et al., 2019; Umeda et al., 2021). Despite these abundant experimental results, the efficacy of the drug in neurodegenerative disorders has been poorly investigated, with contradictory results (Yulug et al., 2018). In a clinical trial testing the efficacy in AD subjects, rifampicin was studied in combination with doxycycline (Molloy et al., 2013). The drugs were well tolerated but the analysis of efficacy did not confirm the positive indications previously reported (Loeb et al., 2004). However, a better selection of patients, earlier treatment and more accurate study design, including other drug combinations, might boost the possibility of a positive readout.

Diosmin, a well-known natural derivative flavonoid, offers diverse neuroprotective activities: in 3×Tg AD model oral diosmin reduced the cerebral accumulation of AβOs and improved abnormal tau pathology, with positive consequences in behavioral tests (Sawmiller et al., 2016). In the same study the authors showed that diosmetin, the biologically active form of diosmin, reduced Aβ generation, tau-hyperphosphorylation, neuroinflammation, as well as γ-secretase and GSK-3 activities *in vitro*, indicating that the biological effects of oral diosmin may be mediated by this metabolite (Sawmiller et al., 2016). More recently, oral diosmin in a rotenone-induce model of PD prevented motor impairment, weight loss, histological damage, and significantly inhibited the rotenone-induced decrease in tyrosine hydroxylase expression (Habib et al., 2022). Like doxycycline, diosmin in a micronized nutraceutical formulation has been successfully used in various pathological conditions (Gerges et al., 2022). Diosmin has an excellent safety profile and was well tolerated, so the clinical studies with diosmin and its various formulations have laid the groundwork safety data for clinical trial in neurodegenerative disorders (Sawmiller et al., 2016).

Natural-based complex polyphenols have been proved to inhibit the formation of AβOs and reduce neuroinflammation in AD experimental models (Tomaselli et al., 2019). Another flavonoid glycoside, rutin, inhibited tau aggregation and TauOs-induced cytotoxicity. In a Tau-P301S mouse model of tauopathy rutin lowered pathological tau levels, regulated tau hyperphosphorylation by raising PP2A levels, suppressed gliosis and neuroinflammation by downregulating the NF-kB pathway, prevented microglial synapse engulfment, and rescued synapse loss in mouse brains, resulting in significant improvement of cognition (Sun et al., 2021b). In a specific study rutin affected Aβ clearance by raising the expression levels of phagocytosis-related receptors in microglia. Rutin also induced a metabolic switch from anaerobic glycolysis to mitochondrial oxidative phosphorylation, which could provide microglia with sufficient energy for Aβ clearance. Thus, rutin might attenuate neuroinflammation, ameliorating synaptic plasticity impairment, and even reversing spatial learning and memory deficits (Pan et al., 2019).

As described by Tikhonova et al. (2022) disease-modifying intervention requiring multifactorial therapy aimed at various pathogenic hubs is a novel trend regarded as a promising strategy for neurodegenerative disorders. The examples reported here indicate several molecules which starting from the anti-oligomeric activity could interfere with other pathogenetic pathways, useful to work out therapeutic strategies with appropriate trial design.

Several other inflammatory pathways are involved in neurodegenerative disorders activated in part by oligomers, including inflammasome (Guan and Han, 2020; Ravichandran and Heneka, 2021). The stimulation of TLRs is part of the activation of the NOD-like receptor pyrin domain containing 3 (NLRP3) inflammasome, a multiprotein complex mainly located in the CNS (Heneka et al., 2013). Clinical data and animal studies indicate that Aβ deposits can cause inflammasome activation (Guan and Han, 2020). Aβ activates microglial cells to produce IL-1β, which is a major outcome of NLRP3 inflammasome activation (Lamkanfi, 2011). Levels of IL-1β level are significantly elevated in the brain tissue, CSF, and peripheral blood of AD patients (Tschopp and Schroder, 2010). Previous studies showed the activation of the NPL3 inflammasome by Aβ fibrils, and recently this activation has been shown *in vitro* by AβOs (Lučiūnaitė et al., 2019). Primary microglial cells were treated with AβOs and NLRP3 inflammasome activation, was determined by caspase-1 cleavage, IL-1β production, and apoptosis-associated speck-like protein containing a CARD speck formation (ASC). The NLRP3 inflammasome inhibitor MCC950 completely inhibited the immune response induced by AβOs (Lučiūnaitė et al., 2019). In cell-free conditions Nakanishi et al. (2018) showed a direct interaction between AβOs, and NPL3 inflammasome through the adopter protein ASC.

NPL3 inflammasome together with TLR-2 has been involved in the protective effect of minocycline against the toxic effects of ICV AβOs in mice. MInocycline neutralized memory impairment and microglial activation induced by AβOs. This last effect was associated with a reduction in TLR-2 content, its adapter protein MyD88, and the levels of the protein NLRP3, which is indispensable in the assembly of inflammasome (Garcez et al., 2019). Treatment with MCC950, an inflammasome inhibitor, antagonized several detrimental effects induced by chronic infusion of AβOs (Fekete et al., 2019). MCC950 attenuated AβO-evoked microglial reactivity, restored the expression of neuronal inhibitory ligands, and abolished memory impairments. MCC950 also eliminated AβO-invoked reduction of serum IL-10 (Fekete et al., 2019).

These studies point to the possible roles of the NLRP3 inflammasome in the pathogenesis of AD and offer the possibility of an NLRP3 inhibitor becoming a potential molecular target for improving AD-related symptoms and slowing AD progression at the neuroinflammatory level (Guan and Han, 2020). The signal transducer of transcription factor-3 (STAT3) belongs to the family of cytoplasmic factors activated by phosphorylation through the Janus kinases (JAK), JAK2/STAT3 pathway is induced by cytokines and in turn regulates several other transcription factors linked to inflammation (Cheon et al., 2011). STAT3 has been proposed having a crucial role in astrocyte activation and modulation of this pathway by overexpressing endogenous inhibitor Suppressor Of Cytokine Signaling 3 (SOCS3) was tested in APP/PS1dE9 mouse model of AD. The treatment, specifically targeted on astrocytes, normalized their activation and mainly restored the histopathological alteration observed in the animal model (Reichenbach et al., 2019).

Similar results were found by Gao et al. (2022) with daphnetin, a natural coumarin derivative and inhibitor of various kinases, in APP/PS1 mice. Daphnetin markedly reduced the expression of glial fibrillary acidic protein and the upstream regulatory molecule- phosphorylated STAT3 in APP/PS1 mice, and mainly inhibited the phosphorylation of STAT3 at Ser727 to lower GFAP expression in a LPS-activated glial cell model (Gao et al., 2022).

Hippocampal microinjections of AβOs have achieved a robust increase in the expression of JAK/STAT3 in glial cells that correlate with neuronal dysfunction (Toral-Rios et al., 2020). *In vitro* results show the potential involvement of STAT3 in PD pathogenesis too (Wang and Liu, 2022).

In primary microglia isolated from wild-type mice α-synOs activated NLRP3 inflammasome via TLR2 and TLR5 ligation, acting on different signaling checkpoints. NLRP3 inhibition by the selective inhibitor CRID3 sodium salt and NLRP3 deficiency improved the overall clearance of α-synOs. In contrast with previous evidence from Gustot et al. (2015) that showed an activation of the NLRP3 inflammasome restricted to α-syn in fibrillary form, these data indicate that α-synOs activate microglial NLRP3 inflammasome, compromising its degradation, which can be prevented by NLRP3 inhibition (Scheiblich et al., 2021). Microglia from adult mice show a phagocytic deficiency for α-synOs and increased TNF-α release, demonstrating that α-synOs induced an inflammatory response (Bliederhaeuser et al., 2016; Du et al., 2022; Lai et al., 2022).

The activation of NLRP3 inflammasome in PD has also been related to peripheral inflammation including the gut-brain axis (Hu et al., 2022). An age-dependent increase of aggregation forms of α-syn has been in the intestinal level, however, this increase is simultaneous with the similar effect in the midbrain. MPTP treatment in a type 2 diabetes (T2D) model increases raises the levels of α-synOs in both pancreas and midbrain, resulting in IL-1β secretion via NLRP3 activation, and ultimately exacerbates the loss of DA neurons (Wang et al., 2014).

## Oligomers and prion protein

The central role of oligomers in the neurodegenerative disorders, independently of the original monomeric sequence, Aβ, α-syn, prion or tau, was based on a direct interaction with neurons. The initial evidence of neurotoxicity employed cell lines and primary neurons, then later the synapses were taken as the main target, and finally the deleterious effects of oligomers were associated with glial activation (Cardinale et al., 2021). The activation of inflammatory pathways by oligomers might be due to glial reactivity induced by neurodegeneration, and the release of inflammatory factors from neurons may activate a vicious circle with deleterious consequence. However, a direct interaction with glial cells is also possible with production of cytokines and other factors which in turn, induce cell proliferation and inflammation.

At the moment both mechanisms can be considered compatible with the neuropathological evidence and experimental findings although, as illustrated above, the ability to antagonize behavioral deficits and neuronal dysfunction associated with oligomers by specific intervention on inflammatory pathways (Stéphan et al., 2003; Balducci et al., 2017; La Vitola et al., 2018; Reichenbach et al., 2019; Gao et al., 2022) obliged us to consider the needful for glial activation in the pathological scenario. The present review illustrates the basis of the biological interaction of oligomers with cell membranes, specific and unspecific, mainly oriented on neurons, however, similar and innovative investigations will be required to establish primary and secondary involvement of glial reactivity in relation to oligomer diffusion and pathological activities.

In the last two decades numerous investigations have looked at the biological effects of oligomers and some aspects need to be considered in an analysis of the results. The biological activities can be triggered essentially by three mechanisms: the interaction with specific entities on cell membranes (Jarosz-Griffiths et al., 2016; Das and Chinnathambi, 2021; Limegrover et al., 2021); unspecific membrane perturbation (Evangelisti et al., 2016; Skamris et al., 2019; Camilleri et al., 2020) and the ability to form a pore channel inside the membrane (Benilova et al., 2012; Stöckl et al., 2013; Lasagna-Reeves et al., 2014; Ghio et al., 2019; Farrugia et al., 2020). These basic mechanisms involving oxidative stress, mitochondrial alterations, glial activation and glutamate receptors are common to virtually all oligomers, regardless of the initial misfolded protein sequence (Kayed et al., 2003; Roberts et al., 2015; Ugalde et al., 2019); other more specific aspects are associated with sequence, size and conformation (Sengupta et al., 2016).

The post-synaptic toxicity of AβOs involves an interaction with NMDA receptors (Shankar et al., 2007; Xia, 2010), triggering a greater contribution of calcium-permeable AMPA receptors, with the ultimate outcome of inhibition of synaptic plasticity and the induction of memory impairment through the prevention/abolition of new dendritic spine formation, where new memories are stored (Chidambaram et al., 2019). Basal synaptic transmission through NMDA receptors is also activated by α-synOs, affecting calcium current and AMPA receptors. Slices treated with α-synOs were unable to respond with further potentiation to theta-burst stimulation, leading to impaired LTP (Diógenes et al., 2012). An interaction with GluN2-NMDA receptors by α-synOs in striatal spiny projection neurons has also been shown, causing visuo-spatial learning deficit (Durante et al., 2019).

Specific entities mediating the neurotoxic activities of oligomers have been investigated in numerous experimental models (Jarosz-Griffiths et al., 2016); together with NMDA receptors and several other elements including APLP1, nicotinic receptor and RAGE, cellular prion protein (PrP*^c^*) has been proposed as mediating the toxic effect of AβOs. Human PrP*^c^* is a 253-residue long precursor polypeptide chain. Its post-translational modifications include removal of the N-terminal 22-residue signal sequence, removal of 23 C-terminal residues, formation of one disulfide bridge (Cys179-Cys214), glycosylation of two asparagine residues (Asn181, Asn197), and binding of a glycosylphosphatidylinositol (GPI) anchor. The N-terminal domain of PrP*^c^* is intrinsically disordered, although it contains four octapeptide repeats with a β-turn or polyproline secondary structure. The C-terminal domain consists of three α-helices and two antiparallel β-sheets. PrP*^c^* is located mainly in the plasma membrane of neuronal cells attached by the GPI anchor, and can act as a receptor or transducer from external signaling. Besides the essential pathological role when converted to a β sheet-rich insoluble conformer (PrP*^sc^*) in TSE, information on the physiological role of by PrP*^c^* is still scant. In the original investigation by Laurén et al. (2009) the affinity of prion protein for AβOs emerged from an unbiased screening using an expression cloning technique in COS-7 cells to identify the AβOs binding site. The binding of PrP*^c^* to AβOs was specific since monomeric and fibrillar forms did not recognize PrP*^c^*, in primary hippocampal cells the biotinylated form of AβOs recognized neurons, and the signal in cultures from PrP^0/0^ mice was reduced but not nullified. In contrast, in cell lines transfected with PrP*^c^* cDNA a new binding for AβOs emerged. The LTP inhibition by AβOs recorded in hippocampal slices was substantially absent in brain slices from PrP^0/0^ mice (Laurén et al., 2009).

These noteworthy results immediately attracted interest in our lab where we had the necessary tools to investigate the functional consequence of the surprising AβOs-PrP*^c^* interaction. ICV injections of synthetic AβOs followed by a behavioral test to verify neuronal dysfunction induced by oligomers was an optimal condition to check for alterations caused by the absence of PrP*^c^*. We compared the effects of AβOs in wild-type and PrP^0/0^ mice, but the detrimental effects in the two conditions were indistinguishable, and memory loss was caused equally with or without PrP*^c^*. Similarly, *in vitro* studies testing AβOs neurotoxicity using primary hippocampal cultures from wild-type or PrP*^c^* deficient mice did not show any differences.

Since differences from the original results might have originated from different AβO preparations we compared AβOs obtained by our routine procedure with those obtained with method described by Laurén et al. (2009), but found no substantial differences in the results. Furthermore, although the memory dysfunction was not influenced by PrP*^c^*, surface plasmon resonance enabled us to confirm a high-affinity interaction between AβOs and PrP*^c^* (Balducci et al., 2010). Since these initial investigations ample data has accumulated supporting one or the other point of view (Calella et al., 2010; Kessels et al., 2010; Cissé et al., 2011; Freir et al., 2011; Resenberger et al., 2012; Rushworth et al., 2013; Serpa et al., 2021).

One possible interpretation of the contradictory results is the complexity of AβOs synaptotoxicity involving the neuronal membrane at various levels and many other elements. PrP*^c^* might possibly be involved in specific conditions, probably as a consequence of sequestration of AβOs rather than a functional activity directly related to the protein (Forloni and Balducci, 2011); this hypothesis was supported by Younan et al. (2013). The model illustrated in our commentary was reminiscent of that proposed by Benilova and De Strooper (2010) and Purro et al. (2018) where PrP*^c^* is one of the numerous elements capable of binding AβOs with different affinity (Li and Selkoe, 2020). Smith et al. (2019) analyzed this aspect using a cell-based assay and several Aβ species: multiple receptor candidates were expressed on the cell surface and the capacity to bind Aβ was compared. Based on the affinity for AβOs the results indicated a prominent role of PrP*^c^*, though it is also possible that the high affinity is not essential to identify the receptors with functional relevance in AD pathogenesis: this calls for specific investigation.

Strittmatter’s group pursued the investigation on the potential role of PrP*^c^* mediating the neurotoxic effect of AβOs, showing that breeding APP/PS1 transgenic mice with PrP^0/0^ mice strongly reduced the memory impairment without affecting Aβ deposition (Gimbel et al., 2010). The same group later showed that Fyn kinase mediates signal transduction downstream of the AβOs-PrPc interaction (Um et al., 2012) in a receptorial complex involving the metabotropic glutamate receptor, mGluR5 (Um et al., 2013). This led to a proposed clinical therapeutic approach to AD (Nygaard et al., 2015), and in a randomized clinical trial Fyn kinase inhibitor AZD0530 was relatively well tolerated but had no positive effects on AD progression (van Dyck et al., 2019).

The AβOs-PrP*^c^* interaction has also been observed in the therapeutic perspectives from a different point of view. The high affinity of AβOs for the N-terminal domain of PrP*^c^* can be exploited to inhibit the biological activity of AβOs by peptides that bind this sequence and inhibit the toxic effect (Nieznanski et al., 2012; Fluharty et al., 2013; Williams et al., 2015).

Although the role of PrP*^c^* as acceptor/receptor of AβOs in the pathogenesis of AD remains to be clarified (Purro et al., 2018), a similar interaction has been proposed for α-synOs (Ferreira et al., 2017). The impairment of LTP induced by α-synOs was blocked in Prnp null mice and rescued by PrP*^c^* blockade. Similar to AβOs, α-synOs formed a complex with PrP*^c^* that induced the phosphorylation of Fyn kinase via mGluR5; whereas the blockade of mGluR5-evoked phosphorylation of NMDAR rescued synaptic and cognitive decline (Ferreira et al., 2017). The clustering of α-synOs and PrP*^c^* was also proposed by Rösener et al. (2020), but in our hands the neurotoxic activity and the ability to induce glial reactivity of α-synOs was not altered by the elimination of PrP*^c^* (La Vitola et al., 2019). In primary hippocampal neuronal cultures from wild-type or PrP^0/0^ mice the neurotoxicity induced by micromolar concentrations of α-synOs was very similar, and the memory impairment induced by ICV α-synOs was comparable in both conditions. The increase of GFAP and IBA-1 immunostaining in hippocampus induced by α-synOs was also indistinguishable in wild-type or PrP null mice, showing that astroglial and microglial activation was independent of PrP*^c^*. Furthermore, in contrast to AβOs, surface plasmon resonance did not show up any physical affinity between α-synOs and PrP*^c^* (La Vitola et al., 2019). Besides minimal methodological differences in recombinant α-syn purification and oligomer preparation, no obvious evidence justifies these contradictory results. A role of prion protein in α-synOs intercellular transport and pathological transmission has been recently hypothesized (Urrea et al., 2018; Rösener et al., 2020) but the link with the direct binding of α-synOs to PrP*^c^* still needs further investigation (Legname and Scialò, 2020; Thom et al., 2021).

The key role of PrP*^c^* as a ligand of toxic proteins in neurodegenerative processes has been extended to the biological effects of TauOs. Ondrejcak et al. (2018) reported that intracerebral application of soluble recombinant tau or soluble tau extracted from AD brain inhibited LTP in mice, and anti-PrP antibodies antagonized the effect. On the basis of this, the authors indicate PrP*^c^* as essential for tau-mediated neuronal disruption of synaptic plasticity *in vivo.* Along this line, Boutajangout et al. (2021) showed that passive immunization with a new anti-PrP antibody significantly reduced tau pathology, resulting in improved cognitive function in a hTau/PS1 transgenic mouse model of AD.

Although Nieznanska et al. (2021) demonstrated a protective effect of prion protein against tau toxicity in specific experimental conditions, the role of PrP*^c^* as a *central player* in neurodegeneration was reiterated by Corbett et al. (2020). In a complex report, these authors show the binding of PrP*^c^* to α-synOs, AβOs and TauOs, with no clear competition for the same PrP N-terminal domain. Impairment of LTP and structural damage (neuritic dystrophy) by oligomers are absent when PrP*^c^* is ablated, or knocked down, or when neurons are pre-treated with anti-PrP blocking antibodies. Unlike in other preparations, the oligomers were produced by sonication from pre-formed fibrils, and generally the other preparations interrupted the self-aggregation when oligomers are formed before further aggregation, halting it by dilution. This methodological difference, as the authors pointed out, might be essential to influence the oligomers’ binding capacity and helped explain the contradictory results. The authors indicated the *protofibrils*, as the conformation state active on PrP*^c^* but did not show the activities of different oligomer conformations. In the second part of the study water-soluble extracts from AD, DLB and Pick’s disease (PiD) brain induced neurotoxic effects dependent on the presence of specific protein aggregates, and immunoprecipitation of single protein Aβ for AD brain extract, α-syn for DLB and tau for PiD nullified the respective toxicity and the elimination of PrP*^c^* from the neuronal cells (iPS-derived) completely antagonized it; furthermore the extract from neurodegeneration-free brain was not toxic at all. The structure of PrP*^c^* might explain the affinity for oligomeric species although the biological question remains whether a single protein can be crucial for four different neurodegenerative disorders, including prion-related encephalopathies.

## Conclusion

The central role of oligomers, small soluble peptide/protein aggregates, as the main culprit in neuronal dysfunction in virtually all neurodegenerative disorders is now commonly accepted. The proteins involved in *oligomeropathies* differ notably in molecular weight and structure of monomeric form, from 4 KDa of Aβ to 350 KDa of huntingtin. The size of oligomers purified from human brain or biological fluid ranges from a few to hundreds of single molecules, but despite this the seeding/nucleation model of formation works well for all the structures. The different localization of protein deposition – extracellular intracellular or intranuclear – might indicate diverse pathogenic mechanisms; however, several biological mechanisms are shared independently from the localization of the deposits. The mechanism of toxicity, spreading and seeding at experimental levels did not distinguish intra- or extra-cellular aggregates. The neurodegenerative process associated to oligomers was initially supported by *in vitro* neuronal cell death induced by micromolar concentrations of Aβ, but later in various experimental conditions reversible neuronal dysfunction and cognitive decline induced by intracerebral AβO in mice at nanomolar concentrations were shown. Recently the pathological scenario has evolved, with a direct contribution of neuroinflammation and glial cell reactivity stimulated in parallel with neuronal disruption induced by oligomers. There has been similar evolution for a-synO. In our *in vivo* model AβO or a-SynO injected ICV induced memory impairment with microglia and astrocyte activation but – more important – anti-inflammatory drugs completely reversed not only the gliosis, but also the cognitive decline (Stéphan et al., 2003; Balducci et al., 2017; La Vitola et al., 2018). Thus, although the pathological sequence activated by oligomers needs to be clarified inflammation seems essential in the neurodegenerative process as shown by numerous other findings. This concept is summarized in Figure 1.

Biological activation by oligomers follows three main mechanisms, an unspecific interaction with cellular and intracellular membranes, the formation of pore-activating channel-like cationic exchanges and, finally, specific interaction with single entities with acceptor/receptor functions. As illustrated in this review, PrP*^c^* has been proposed as mediating the toxicity of Aβ, α-syn and tau oligomers, and at least for AβO-PrP*^c^* ample data supports this evidence although the functional consequences are still debated. The association of PrP*^c^* with α-synOs and TauOs is a more recent acquisition and other investigations are needed to produce a solid biological hypothesis. In any case the importance of these interactions in the pathogenesis of diseases needs to be proved and the relationship with glial activation is still being sought.

These results, as a whole, orient the therapeutic approaches to neutralize the initial pathological steps involving oligomers in the neurodegenerative process: deposit formation, oligomer circulation, inflammation. The numerous failures reported in AD, synucleinopathies and FTD with disease-modifying therapies have different explanations, treatment timing, selection of patients, wrong therapeutic target, wrong trial design and obviously the diseases complexity. As considered elsewhere (Forloni, 2020) multifactorial diseases call for better characterization of the patients and probably several therapeutic tools. In our investigations doxycycline showed double protective activity – anti-amyloidogenic and anti-inflammatory – in experimental models focused on different oligomer species. Thus a single molecule might combine multiple pharmacological targets and doxycycline repositioning has the advantage of being immediately available for clinical investigations (D’Souza et al., 2020). This multi-target activity is shared by several other molecules and treatments (Dias Viegas et al., 2018; Aboushady et al., 2021; Santamaria et al., 2021; Fantacuzzi et al., 2022), that need to be tested at the clinical level with appropriate trial design.

The biology of oligomers and their physico-chemical characteristics have been extensively studied at experimental level, and although several aspects remain elusive, as illustrated here, the main point for the future of this research is translating the scientific achievements to the clinical stage. The identification of oligomers as diagnostic tools using imaging analysis or biochemical determination in biological fluids, including purified exosomes, appears fundamental, especially in the early stages of the disease. But then too, appropriate therapeutic strategies must be worked out targeting oligomers themselves or biological processes closely associated with their formation, diffusion and biological activities. These are the next challenges for this fascinating area of biomedical research that call for wide range of specialist contributions.

## Author contributions

GF wrote the final version of the review. PL and CB contributed to the elaboration of results and discussion of review content. All authors contributed to the article and approved the submitted version.
